# 

**DOI:** 10.1038/sj.bjc.6600406

**Published:** 2002-01-02


analysis of tumour tissue demonstrated that selective virus replication had occurred.

In the trial reported here, patients with high grade glioma had their tumours surgically resected and $10^5$ pfu of the HSV1716 injected into tissue adjacent to the cavity. This allowed assessment of safety HSV1716 injection into normal brain. Of the twelve patients recruited, seven had recurrent disease, and five were newly diagnosed. Eleven patients received further treatment, either radiotherapy or chemotherapy. No evidence of toxicity associated with HSV1716 has been detected one patient who became ill with pyrexia of unknown origin (six weeks following HSV1716) but with no evidence of systemic herpes simplex infection, clinically or immunologically was given intravenous Acyclovir as a precautionary measure.

Two of the patients with recurrent glioblastoma have died due to disease progression at two and six months post virus injection, and four of the remaining ten patients have shown clinical and radiological evidence of tumour regrowth. One of the newly diagnosed patients who showed evidence of tumour regrowth has undergone further surgical resection. The remaining six patients are presently well at 7 weeks to 9 months post virus administration.

This study has shown HSV1716 into tumour cavity to be a safe approach. Subsequent time to progression data will provide evidence of potential efficacy. The information obtained from this and previous studies allows informed design of planned Phase 2 efficacy trials.

### 6.6

### EVALUATION OF DRUG COMBINATIONS AS A STRATEGY TO ERADICATE QUIESCENT LEUKAEMIC STEM CELLS IN CHRONIC MYELOID LEUKAEMIA

H. Jørgensen*, E. Allan, S. Graham, L. Richmond, J. Godden and T. Holyoake

ATMU, Dept of Medicine, University of Glasgow, Royal Infirmary, Glasgow G31 2ER.

The development of a small molecule antagonist of BCRABL, the fusion oncoprotein that is thought to be causative in Chronic Myeloid Leukaemia (CML), heralds a new era in the treatment of this otherwise refractory disease. However, possible mechanisms of resistance to Glivec® (imatinib mesylate, Novartis, Switzerland) have been described including gene amplification and P-glycoprotein up-regulation. We have previously demonstrated the existence of quiescent (G0), leukaemic (Philadelphia, $Ph^+$), stem ($CD34^+$) cells in all CML samples studied. Moreover, these cells have been shown to be insensitive to Glivec® *in vitro*, even at a concentration 10-fold higher than normally achieved in patient plasma (Graham et al., Blood 2002; 99(1): 335). Indeed, in the presence of growth factors, the percentage recovery of input $CD34^+$ cells in the G0 fraction was significantly greater in the presence of Glivec® than in the absence of drug, suggesting an accumulation of G0 cells, an effect modulated by the drug itself. Further, $Ph^+$ $CD34^+$ G0 cells have been identified by D-FISH in the majority of patients in complete cytogenetic remission following Glivec® treatment who mobilised sufficient $CD34^+$ cells for peripheral blood stem cell harvest. We believe it is imperative to formulate a strategy to eradicate the persistent Glivec®-insensitive G0 stem cell population that retains the theoretical ability to repopulate the disease.

Standard $^3$H-thymidine-uptake cell proliferation assays have been performed using the human $Ph^+$ blast crisis derived cell line, K562, in order to determine the effective concentration range of individual drugs selected to be tested for additive/synergistic activity in combination with Glivec®. Currently, two drugs have been assayed, namely the anti-metabolite cytosine arabinoside (Ara-C), and the novel farnesyltransferase inhibitor (FTI) SCH66336 [Schering Plough]. Both Ara-C and FTI were effective inhibitors of K562 proliferation (IC50s of 0.05M and 10M, respectively). Encouragingly, when tested in combination at their individual IC50, Ara-C plus Glivec® (IC50 = 0.5M) elicited >92% inhibition with respect to no drug control, FTI plus Glivec® produced >94% inhibition of K562 proliferation. In order to determine the effect, if any, of these combinations on the $Ph^+$ $CD34^+$ G0 cell population, methodology developed in our lab, using the vital fluorescent dye, CFSE, to track cell divisions, was implemented. Briefly, $Ph^+$ $CD34^+$ primary CML stem cells are labeled with CFSE and cultured for 72 hours ± test compound(s) in the presence of 5 growth factors. The cells remaining viable ($PI^-$) are then sorted on the basis of CFSE fluorescence intensity and the percentage recovery of input cells can then be calculated for all peaks (undivided and divided). Interestingly, both Ara-C and FTI individually caused an apparently greater accumulation of G0 cells than Glivec® alone. Moreover, Ara-C in combination with Glivec® elicited a further 38% increase in number of viable, leukaemic stem cells than with Ara-C alone. Further experimental compounds will be tested in this system, namely LY294002 (CN Biosciences), PEG-Intron (Schering) 17 AAG and Byrostatin (both NCI, Bethseda. MD).

### 6.7

### COMPARISONS OF PROGNOSTIC FACTORS IN PATIENTS PARTICIPATING IN PHASE I CLINICAL TRIALS WITH CONVENTIONAL CYTOTOXIC DRUGS VERSUS NEW NON-CYTOTOXIC AGENTS

C Han*, J Braybrooke, DT Mackintosh, TS Ganesan, AL Harris, DC Talbot

Cancer Research UK, University of Oxford, Churchill Hospital, Oxford, OX3 7LJ, UK.

**Aims:** Little is known about prognosis of patients entering phase I trials. The objectives of this retrospective study were: (1) to identify prognostic variables for toxicity and survival in patients (pts) participating in phase I clinical trials a single centre; (2) to compare characteristics of pts treated with cytotoxic chemotherapy (CT) and non-cytotoxic drugs (non-CT); (3) to determine whether being treated by non-CT might have any impact on prognosis or toxicity.

**Patients and methods:** Data were collected from 420 (114 CT, 306 non-CT) pts enrolled in 16 phase I trials (5 CT and 11 non-CT trials) in our institution between 1991 and 2000. Pt characteristics: 210M, 210F, median age 56 yrs (22-87), median PS 1 (0-3), median duration of treatment 57 days (2-393). The chi-square test and Mann-Whitney test were used to compare treatment groups. Univariable analyses of survival times were performed using the log-rank test. Cox proportional hazards model was adopted to estimate prognostic factors in overall survival (OS). The Prognostic Index (PI) was generated from data on all 420 pts. A logistic regression model was used to explore predictive variables of toxicity. All studies were approved by the Central Oxford Research Ethics Committee.

**Results:** Overall tumour response: 4.5% (95% CI: 2.7-7.0%); Median OS: 202 days (95% CI: 189-249). Multivariate analysis showed that pts with better PS, high Hb, WBC or LDH in normal range and fewer sites of metastases had significantly better OS. Males, pts with low platelet count, high WBC count or treatment with a non-CT phase I agent had significantly less chance of toxicity. Comparing CT with non-CT treated groups: the CT group had a higher proportion of females ($p < 0.001$), were younger ($p = 0.04$), had better PS ($P=0.02$), were more likely to have liver involvement ($p<0.001$) and have LDH in normal range ($p=0.001$). Pts in the CT group had a longer duration of treatment (median time on study 73 vs 51 days, $p<0.001$), better tumour response (1 CR and 15 PR versus 3 PR, $p<0.001$). Median survival was not significantly different between the CT and non-CT groups (260 vs 192 days, $p = 0.47$). In pts with liver metastases (n = 127) median survival was significantly shorter in the non-CT group (137 vs 228 days, $p = 0.02$).

**Conclusion:** Multivariate analysis supports the view that high LDH or WBC, low Hb, poor PS and a higher number of metastases are independent adverse prognostic variables for survival. Female, treatment with phase I CT drugs, low baseline WBC and high platelet counts are independent predictors of toxicity. Cytotoxic agents, compared to non-CT, are more likely to induce response, although OS is not affected by the class of agent. We conclude that entry into a phase I trial of a non-CT drug is a safe option in this population of usually heavily pre-treated pts. The PI generated from these data could be used to estimate the survival probability for pts entering phase I studies in the future.

### 6.8

### AIM HIGH – ADJUVANT INTERFERON IN MELANOMA (HIGH RISK): THE COSTS.

B.W.Hancock*[1], S.Dixon[2], L.A.Turner[1], K.Wheatley[3], N.Ives[3], M.Gore[4], [1]Weston Park Hospital, Sheffield, [2]School of Health & Related Research, Sheffield [3]University of Birmingham, [4]Royal Marsden Hospital, London

© 2002 Cancer Research UK

© 2002 Cancer Research UK

Approval from the local ethics committee was obtained. Thirty-one patients with confirmed transitional cell carcinoma of the bladder received pimonidazole prior to transurethral resection of the tumour. Sections available from 26 tumours were double stained for pimonidazole (hypoxia) with Ki67 (proliferation), & pimonidazole with CD31/34 (vascularity). Sections were also stained for GLUT 1 and CA9. Sections were analysed with the aid of image analysis software.

The median hypoxic fraction assessed by pimonidazole was 9% (range 0 to 38%). The geographical match and fraction of tumour stained by GLUT 1 and CA9 was very similar to pimonidazole (correlation coefficient 0.86 and 0.76 respectively). Most staining was distant from vessels (median 99μm). Some hypoxia was close to vessels (<40μm), suggesting a perfusion limiting mechanism to oxygen delivery in these areas. Dual staining for pimonidazole and proliferation showed a predominantly inverse relationship between these factors. However, some hypoxic regions of less than 40μm from a vessel had a higher proliferative index than those further away from vessels.

Having confirmed GLUT1 and CA9 as appropriate intrinsic markers for hypoxia, archived paraffin embedded samples of bladder carcinoma from 64 patients treated with ARCON were obtained. Sections were stained for GLUT1, CA9, Ki67 and CD31/34 and analysed as above.

The median age of the group was 72.9 years (range 38 to 88 years). Six patients had T1 disease, 19 had T2 disease, 38 had T3 disease and one had T4 disease. Forty-five tumours were grade 3, 17 were grade 2 and 2 were grade 1. The median follow up time was 27 months.

The median stained fraction for GLUT1 was 6.5% (range 0 to 62%) and for CA9 was 3.5% (range 0 to 67%). Those patients above the median (more hypoxic tumours) for each marker had a statistically significantly worse cause specific survival (GLUT1 $p<0.005$, CA9 $p<0.003$) but not local recurrence or metastasis free survival, analysed with a log rank test. A multivariate analysis, accounting for stage, age, grade, Ki67 index and vascularity confirmed hypoxic fraction as an independent factor in cause specific survival (GLUT1 $p<0.02$, CA9 $p<0.03$).

In conclusion, hypoxia is a significant factor in bladder cancer, and can be quantified with both extrinsic and intrinsic markers. Staining patterns are consistent with diffusion limited and perfusion. limited mechanisms of hypoxia. Proliferation is reduced in hypoxic areas, the greatest effect seen with increasing distance from vessels. In a cohort of patients with bladder cancer treated with ARCON, hypoxic tumours were associated with a significant reduction in cause specific survival.

### 7.4
### A NOVEL PROMOTER-ENHANCER FOR HYPOXIA-SELECTIVE GENE THERAPY.

N Chadderton[1*], RL Cowen[1], KJ Williams[1], PJ Ratcliffe[3], AL Harris[2], IJ Stratford[1], [1]School of Pharmacy and Pharmaceutical Sciences, University of Manchester, Manchester M13 9PL, UK, [2]IMM, John Radcliffe Hospital, Oxford OX3 9DU, UK, [3]Welcome Trust Centre for Human Genetics, Oxford OX3 7BN, UK.

We are developing a cancer gene therapy targeted to hypoxia, a unique feature of solid tumours, which is transcriptionally silent in normal tissues. The efficacy of transcriptional targeting with any specific promoter is directly related to the toxicity of the transgene being used. The development of tightly regulated specific enhancer/promoter elements is pivotal to deliver potent therapeutic genes to provide a clinically relevant treatment modality. Hypoxic response is dependent upon the binding of the transcription factor HIF-1 to its cognate receptor, the hypoxia response element (HRE). Using HREs derived from the mouse phosphoglycerate kinase (PGK) and lactate dehydrogenase (LDH) genes in combination with minimal promoter elements from the tumour associated carbonic anhydrase-9 (CA-9) we have developed a number of novel promoter enhancer combinations tightly controlled by hypoxia.

CA-9 is strongly inducible by hypoxia in a broad range of tumour cells, yielding levels of expression comparable to those achieved with full length SV40. This response is regulated by a HRE within the minimal promoter. The HRE-CA-9 elements were introduced into the pGL3-basic luciferase reporter vector and transiently transfected into a panel of 9 human tumour cell lines. The response of the vectors to air or anoxia (0.002% $O_2$) for 16 and 40 hours was tested. A range of optimal HRE driven SV40 constructs (pGL3-Prom), and CA-9 alone vectors were tested in parallel for comparison. We have consistently seen a 6-fold reduction in spurious background expression following evaluation of the HRE-CA-9 constructs against their HRE-SV40 pairs. We have further enhanced anoxic induction by exploiting the unique positioning of the CA-9 HRE, 4bp from the transcriptional start site, to construct novel hypoxia regulated enhancer and promoter 'fusions'. The replacement of the native CA-9 HRE with an optimal HRE composed of a triplicate LDH HRE ($LDH^{3-}$) resulted in up to a 30-fold enhancement of expression compared to the native CA-9. Whilst maintaining transcriptional silence in air we have achieved levels of induction comparable to our optimized vector, $LDH^{3-}$SV40.

This application of the minimal CA-9 promoter provides both a further degree of hypoxia regulated, tumour-specific expression and an attractive alternative to viral promoters that can be shut down *in vivo*. This data demonstrates the ability of this novel vector to deliver a suitably high level of gene expression for gene therapy.

### 7.5
### HYPOXIA SELECTIVE GENE DIRECTED ENZYME PRODRUG THERAPY

Rachel L. Cowen*1, Kaye J. Williams1, Mohammed Jaffar1, Adam V. Patterson1,7, Brian A. Telfer1, and Ian J. Stratford1.
1school of pharmacy and pharmaceutical sciences, university of manchester, oxford rd., manchester, u.k., 7auckland cancer society research centre, the university of auckland, auckland 1000, N.Z.

The aim of our research is to combine hypoxia driven gene therapy and bioreductive chemotherapy to selectively target therapeutically refractive hypoxic tumours. to this end we have developed a novel synthetic series of indolequinone pro-drugs similar in structure to the clinical bioreductive drug mitomycin c (mmc). upon evaluation of the bioreductive cytotoxicity of this series in aerobic and hypoxic conditions a lead compound was identified 5-aziridinyl-3-hydroxymethyl-1-methylindole-4,7-dione (629). we have compared the potency of 629 and its selectivity for activation by the reducing enzymes cytochrome P450 reductase (P450R) and dt-diaphorase (DTD) using stable cell lines, derived from a panel of human breast carcinoma cell lines, which over-express these enzymes.

| Cell line | Clone | Enzyme induction | $IC_{50}$ (μM) | | HCR |
|---|---|---|---|---|---|
| | | | Air | Anoxia | |
| MDA468 | WT | - | 34.82 | 0.268 | 130 |
| | P450R | 8 | 2.526 | 0.00408 | 619 |
| | DTD | 115 | 0.02 | 0.00446 | 4.9 |
| T47D | WT | - | 11.2 | 0.57 | 19.6 |
| | P450R | 60 | 0.49 | 0.0093 | 52.7 |
| | DTD | 278 | 0.18 | 0.025 | 7.3 |
| MDA231 | WT | - | 25.6 | 2.56 | 10 |
| | P450R | 28 | 2.15 | 0.036 | 59.7 |
| | DTD | 935 | 0.0225 | 0.0133 | 1.7 |

The data above suggests that 629 is preferentially activated through one electron reduction under conditions of low oxygen making it an ideal prodrug for an hypoxia driven GDEPT approach. To activate this novel pro-drug we have constructed adenoviral vectors encoding for constitutive P450R expression (Ad CMV P450R) and two vectors encoding for hypoxia induced P450R expression. These vectors ad PGK P450R and Ad LDH P450R contain hypoxia responsive promoters derived from phosphoglycerate kinase and lactate dehydrogenase a, respectively. We have demonstrated that all three viruses infect the breast tumour cell lines with high efficiency (40-80% transduction with moi 300). Using ad CMV P450R a 13 to 40 fold over-expression of P450R can be achieved compared to uninfected cell levels. We have also demonstrated a 5-10 fold increase in P450R levels specifically restricted to cells infected with Ad PGK P450R and Ad LDH P450R that have been exposed to hypoxia. These vectors are currently being evaluated

© 2002 Cancer Research UK

for their ability to sensitise hypoxic tumours to both 629 and MMC *in vitro* and *in vivo*.

### 7.6
### VIRUS-DIRECTED ENZYME PRODRUG THERAPY (VDEPT): CLINICAL TRIALS WITH ADENOVIRAL NITROIMIDAZOLE REDUCTASE (Ad-ntr)

Daniel H Palmer[*, 1], Vivien Mautner[1], Darius Mirza[2], John Buckels[2], Simon Olliff[2], Diana Hull[2], Andrew Mountain[4], Peter Harris[4], Hakim Djeha[4], John Ellis[4], Mark Horne[4], Simon Hill[4], Christopher Wrighton[4], Peter Searle[1], David J Kerr[3] , Lawrence S Young[1], Nicholas E James[1]
1.Cancer Research UK Institute for Cancer Studies, University Of Birmingham, UK; 2. Queen Elizabeth Hospital, Birmingham, UK; 3. Department Clinical Pharmacology, University of Oxford, UK; 4. COBRA Therapeutics Ltd, Keele, UK.

The bacterial enzyme nitroreductase (ntr) converts the prodrug, CB1954, to a highly toxic, short-lived, bifunctional alkylating agent. Since there is no human homologue of ntr, a VDEPT approach utilising ntr / CB1954 is feasible.

We have constructed a replication deficient adenovirus (Ad-ntr) that expresses ntr from the non-selective CMV promoter. Using Ad-ntr we have demonstrated in vitro activity, with a marked bystander effect, in a range of tumour cell lines. We have also demonstrated in vivo activity in subcutaneous xenograft models following intratumoural injection of Ad-ntr followed 48 hours later by intravenous CB1954.

A phase I trial to determine the maximum tolerated dose of Ad-ntr was undertaken in patients with hepatic metastatic colorectal cancer awaiting hepatic resection. Escalating doses of virus ($1.05x10^8$ – $1.05x10^{11}$) have been administered into the tumour percutaneously under ultrasound guidance. Three patients were treated at each dose level and additional patients were recruited if there was significant (>grade 2) toxicity. Liver resection was performed 48-96 hours after virus injection following which the tumour was examined for evidence of ntr expression immunohistochemically.

11 patients have entered this study (virus particle dose of $10^8$, n=3; $10^9$, n=3; $10^{10}$ , n=4; $10^{11}$ , n=1). Toxicity has been minimal (1 patient had systemic flu-like symptoms) and there is a dose-related increase in PCR aided detection of viral DNA in venous blood. Neutralising antibodies to Ad-ntr are formed but there is significant inter-patient variation in kinetics and antibody class. Ntr is detected in resected tumours (1-3% of cells) with a dose-dependent increase in cell positivity. We have not yet reached our target percentage of cancer cell population staining positively for ntr, at which stage we will co-administer CB1954 at a dose ascertained in an earlier phase I and pharmacokinetic study (24mg/m$^2$ IV).

Ad-ntr has also entered clinical trials of intratumoural injection in head and neck and prostate cancer.

### 7.7
### DIRECT INJECTION OF ADENOVIRUS FOR ENZYME PRODRUG THERAPY OF PROSTATE CANCER

J.G. Young[1], H.Y. Leung[3], P.F. Searle[1], V. Mautner[1], A Howie[2], M.Horne[4], J.Ellis[4], J.Gilbert [4]; D.E. Neal[3], P. Harris[4], A.P. Doherty[2], D.M.A. Wallace[2], N.D. James[1]*. (1). CRC Institute for Cancer Studies, University of Birmingham (2). University Hospital Birmingham, (3). Freeman Hospital, Newcastle upon Tyne, (4). Cobra Therapeutics, Keele.

**Introduction:** the ease of direct access to the prostate by transperineal, transurethral and transrectal routes makes locally targeted therapies for prostate cancer a viable option. We are undertaking a clinical trial of gene therapy administered by direct intraprostatic injection of adenovirus followed by intravenous injection of prodrug. The transgene *e. Coli* nitroreductase (ntr) has been inserted into a replication deficient adenoviral gene therapy vector, called ctl102, under the control of the cytomegalovirus immediate early promoter. Ntr converts the weak monofunctional alkylating agent 5-(aziridin-1-yl)-2,4-4-dinitrobenzamide (cb1954) into a highly cytotoxic bifunctional alkylating agent that kills both replicating and non-replicating cells. In preclinical models, intratumoural ctl102 injection of subcutaneous pc-3 tumours in nude mice, followed by systemic cb1954 resulted in inhibition of tumour growth. The study involves: (1) a marker study to optimise a clinical protocol for *in vivo* prostatic ntr expression, (2) investigation of the effects of direct injection of escalating doses of ctl102, with cb1954, into locally recurrent prostate cancer. Patients & methods: virus tropism for the prostate was verified by ex vivo infection of thin slices of freshly resected prostate tissue with a related virus expressing green fluorescent protein (ad-gfp) in place of ntr. The trial is divided into two parts; firstly the marker study involves a dose escalation phase i investigation in patients scheduled to undergo radical prostatectomy. Secondly, we will investigate a combination of virus and prodrug in patients with locally recurrent disease. The patients will undergo intraprostatic injection of ctl102 under transrectal ultrasound control in escalating doses, starting from $10^{10}$ adenoviral particles, rising to $10^{12}$ particles, in cohorts of three patients, with subsequent radical prostatectomy at 48-72 hours post injection. Ntr expression in the resected tissue is assessed by immunohistochemistry. The dose escalation is aimed at establishing the dose of ctl102 at which significant transgene expression is detected as well as conventional phase i toxicity endpoints. **Results:** in vitro culture of fresh prostate tissue with adenovirus expressing gfp revealed diffuse expression of transgene throughout glandular & stromal elements. 2 patients have now been treated with ctl102 in the marker study with $10^{10}$ particles of adenovirus with no serious adverse reactions. Trus images following injection demonstrate hyperechoic virus suspension disseminating slowly throughout the injected prostate lobe. Immunohistochemistry of prostate resected from the first patient has revealed transgene expression, apparently confined to glandular epithelium. **Discussion:** direct intraprostatic injection appears to be feasible and safe. Early results suggest that injected material disseminates via the prostatic ducts, the tissue of origin for prostate cancer. Evidence of virus infection and expression of the ntr protein has been seen at the lowest dose level and dose escalation is planned. Once nr expression at pre-determined levels has been demonstrated, the trial will proceed to administration of both virus and prodrug in locally recurrent cases.

### 7.8
### RADIATION-ACTIVATED PRODRUGS: HYPOXIA-SELECTIVE RADIOLYTIC RELEASE OF POTENT CHLOROMETHYLBENZINDOLINE (CBI) CYTOTOXINS FROM NOVEL NITROARYLMETHYL QUATERNARY (NMQ) PRODRUGS

Kriste A.G.*, Tercel M., Stribbling S.M., Botting K.J and Wilson W.R., Auckland Cancer Society Research Centre, The University of Auckland, Private Bag 92019, Auckland, New Zealand.

Hypoxia is a major negative prognostic factor in radiation therapy. We have previously suggested the possibility of activating non-toxic prodrugs to potent cytotoxins by reducing them with ionizing radiation in the absence of oxygen. Reductive fragmentation of the nitroarylmethyl quaternary (NMQ) ammonium prodrugs of mechlorethamine (HN2) has been demonstrated by radiation under anoxic conditions, but the prodrugs are not sufficiently stable *in vivo*, and the released nitrogen mustard cytotoxin is not sufficiently potent for this mechanism to be therapeutically useful at the radiation doses used clinically. The aim of this study was to develop novel NMQ prodrugs capable of releasing significantly more potent chloromethylbenzindoline (CBI) cytotoxins on radiolytic reduction. Novel 5-amino- or 5-hydroxy-*seco*-CBI cytotoxins were synthesized and demonstrated to have $IC_{50}$ values (following 4 hr exposure) of ca.1 nM in a panel of human tumour cell lines. The corresponding 4-nitroimidazolyl (4NI) and 2-nitropyrrolyl (2NP) NMQ salts were 20 to 200-fold less potent, demonstrating significant deactivation in the prodrug form. When cells were exposed under anoxic conditions, $IC_{50}$ values were 1.4 to 8-fold lower, indicating modest bioreductive (enzymatic) activation under anoxia. The hydroxy-NMQ-CBI prodrugs had superior stability to the amino analogues in culture medium, as assessed by LC/MS, and were investigated further. When irradiated in anoxic formate buffer, these NMQ-CBI prodrugs (20 μM) released their hydroxy-CBI effectors efficiently (HPLC analysis; $G$ values 0.45-0.54 μmol $J^{-1}$), indicating a stoichiometry close to one reducing equivalent. The 4NI and 2NP prodrugs (5 μM) were irradiated in anoxic human plasma and effector release was assessed by bioassay. These experiments demonstrated a 10 to 15-fold increase in cytotoxicity at 18 Gy, with an estimated $G$ value for hydroxy-CBI release of ca 0.02-0.08 μmol $J^{-1}$.The extravascular diffusion properties of the 4NI and 2NP prodrugs are currently being assessed in a multicellular layer tumour model, while initial studies in mice indicate that the MTD values for the NMQ prodrugs are more than 10-fold higher than for the CBI effector,

© 2002 Cancer Research UK

suggesting adequate metabolic stability *in vivo*. An investigation of their activity against hypoxic cells in HT29 tumors, in combination with radiation, is in progress.

This work was supported by the Wellcome Trust and the New Economy Research Fund of New Zealand.

### 8.1

### NOVEL DIRECT TARGETS FOR THE CONTROL OF GROWTH BY c-MYC

Natividad Gomez-Roman[1], Carla Grandori[2], Robert N. Eisenman[2] and Robert J. White[1]*

[1]Institute of Biomedical and Life Sciences, Division of Biochemistry and Molecular Biology, University of Glasgow, Glasgow, G12 8QQ, UK

[2]Division of Basic Sciences, Fred Hutchinson Cancer Research Center, Seattle, Washington, 98109-1024, USA

The proto-oncogene product c-Myc lies at the interface between cell growth and division, impacting directly on both these processes. Indeed, Myc can stimulate cell growth (mass increase) independently of cell cycle effects. Since the bulk of a cell's dry mass is protein, growth is determined by the rate at which protein accumulates. Accordingly, Myc has been shown to stimulate the production of the protein synthetic apparatus. Activation of Myc causes increased synthesis of translation factors, ribosomal proteins and rRNA (1-3). We have found that c-Myc also activates the transcription of tRNA genes by RNA polymerase (pol) III. Thus, tRNA transcripts are rapidly induced by Myc in primary human fibroblasts. Furthermore, the expression of pol III products is significantly reduced following targetted disruption of the c-Myc gene. These effects reflect the direct action of c-Myc on the pol III machinery. Chromatin immunoprecipitation reveals the presence of c-Myc at tRNA genes in vivo. Moreover, c-Myc binds to the pol III-specific transcription factor TFIIIB. Inducing tRNA production is likely to contribute to the activation of protein synthesis that enables Myc to stimulate cell growth.

1. Elend, M. & Eilers, M. (1999). Curr Biol 9, R936.
2. Schmidt, E.V. (1999).Oncogene 18, 2988.
3. Grandori, C., Cowley, S.M., James, L.P. & Eisenman, R.N. (2000). Annu Rev Cell Dev Biol 16, 653.

### 8.2

### DIFFERENTIAL EXPRESSION OF FIBROBLAST GROWTH FACTOR RECEPTOR SUBSTRATES IN HUMAN MALIGNANCIES

SY Ng*, CN Robson, DE Neal, HY Leung. Prostate Research Group, School of Surgical and Reproductive Sciences, Medical School, University of Newcastle, Newcastle upon Tyne, NE2 4HH.

***Introduction***

FGF receptor substrate (FRS), a membrane-anchored adapter protein targets signalling molecules to the plasma membrane in response to FGF stimulation. The two FRS isoforms, FRS2$\alpha$ and FRS2$\beta$ (commonly referred to as FRS-2 and –3), are structurally similar and share 49% amino acid sequence identity. FRS is required for FGF-induced MAPK activation. We examined the levels of FRS-2 and -3 expression in a panel of human cancer cell lines and correlated their expression to FGF induced MAPK activation.

***Materials and Methods***

FRS-2 and -3 expression was detected by RT-PCR using specific primers. The following cell lines were included: prostate (LNCaP, PC3 and DU145), breast (MCF7), bladder (T24, J82 and RT112), melanoma(M2), colorectal (HT29) and osteoblastic (U2OS). FRS2 protein expression was further quantified following Western blotting. MCF7, LNCaP and J82 cells were selected for MAPK activation study, and the effect of FGF1 on ERK1/2 activation was quantified using phosphospecific antibody.

***Results***

FRS2 transcript was widely expressed and represents the predominant form of FRS expressed: 6/8 and 3/8 cell lines with detectable FRS-2 and FRS-3 expression, respectively. On Western blotting, LNCaP (prostate) and MCF7 (breast) cells expressed FRS2 at high level as a 90kD protein. T24 (bladder), M2 (melanoma), PC3 and DU145 (both prostate) expressed moderate levels of FRS2. FRS2 was expressed at low level in J82 (bladder) and HT29 (colorectal). The U2OS (osteoblastic) and RT112 (bladder) cells did not have any detectable levels of FRS2 expression. Using densitometry, corrected to $\alpha$-tubulin expression, there was a 16 fold difference in FRS-2 expression between MCF7 and J82 cells. Consistent with the observed pattern of FRS2 expression, FGF1 induced pERK1/2 level was highest in MCF7 cells, followed by LNCaP and was lowest in J82; their corresponding ratio of activation were 27, 13 and 7, when compared to serum starved controls. Of note, the three cell lines (MCF7, LNCaP and J82) did not have significant FRS-3 expression.

***Conclusion***

FRS-2 appears to be the predominant form of FRS expressed in human malignancies. FRS-2, in the absence of FRS-3 expression, is adequate for FGF induced MAPK activation and the levels of FRS-2 expression correlates with the degree of FGF induced MAPK activation.

### 8.3

### INVESTIGATING THE ROLE OF POLY(ADP-RIBOSE) POLYMERASE-1 IN THE RESPONSEOF GLIOMA CELLS TO LOW DOSES OF IONISING RADIATION.

Anthony Chalmers[1]*, Mick Woodcock[1], Brian Marples[2], Mike Joiner[2] Peter Johnston[1].

[1]Gray Cancer Institute, Mount Vernon Hospital, Northwood, Middlesex, HA6 2JR.

[2]Karmanos Cancer Institute, Wayne State University, Detroit, MI 48201, USA.

**Introduction:** The terms low-dose hyper-radiosensitivity (HRS) and increased radioresistance (IRR) describe the dual phenomena observed in many mammalian cell systems where small acute doses of radiation below ~50 cGy are more lethal per unit dose than higher doses up to ~2 Gy. There is evidence that HRS and IRR may reflect dose-dependent changes in DNA repair mechanisms. Recent work also suggests that the extent of HRS varies according to cell-cycle phase, being most marked in G2. Poly(ADP-ribose) polymerase-1 (PARP-1) is an abundant, highly conserved nuclear enzyme that rapidly binds to and is activated by DNA single and double strand breaks leading to recruitment and modulation of DNA repair processes. Evidence for a key role for this enzyme in the cellular response to radiation derives from PARP-1 knockout mice, and chemical and molecular inhibitors of PARP-1. Inhibition of PARP-1 was associated with sensitisation to radiation doses of 2 Gy and above (sensitiser enhancement ratio ~1.5) (ref). To date, however, the contribution of PARP-1 to the low-dose radiation response has not been extensively investigated. We are studying the effect of new, highly potent and specific inhibitors of PARP-1 on low-dose clonogenic survival of human glioma cell lines that display markedly differing degrees of HRS and IRR, with the dual aims of elucidating the role of PARP-1 in these phenomena, and investigating the potential for therapeutic symbiosis between PARP-1 inhibitors and an ultrafractionated radiation schedule.

**Methods:** Clonogenic survival of T98G (HRS+) and U373 (HRS-) human glioma cells following low-dose irradiation in the presence of the following chemical inhibitors of PARP-1 are being determined using a highly accurate fluorescence-activated cell sorting (FACS) clonogenic assay: 3-aminobenzamide, NU1025, 4-amino naphthalimide (4-ANI) and PJ34. All inhibitors have been tested at a range of concentrations to establish the optimum dose for modifying radiation response with minimal direct cytotoxicity. Further studies will investigate the effect of active drugs on the low-dose radiation responses of cells sorted according to cell-cycle phase.

**Results:** Preliminary studies utilising the first generation PARP-1 inhibitor 3-AB have demonstrated a modification of the low-dose radiation responses of both T98G and U373 cell lines that is complicated by the cytotoxic action of the chemical. NU1025 has exhibited an unexpected protective effect at low radiation doses (characterised by an enhancement in cell plating efficiency) that is being investigated.

**Discussion:** Exploiting the potency and specificity of new PARP-1 inhibitors will elucidate the role of PARP-1 in HRS/IRR, enabling assessment of the potential for a therapeutic interaction of such inhibitors with an ultrafractionated radiation schedule in the treatment of gliomas.

© 2002 Cancer Research UK

8.4

**Nuclear BAG-1 expression predicts survival from breast cancer, and potentiates oestrogen dependEnt transcription.**

Ramsey I Cutress*, Paul A Townsend, Lynn Wood, Matthew Brimmell, Anna Maison, Ron Lee, Mark A Mullee, Peter WM Johnson, Gavin T Royle, Adrian C Bateman and Graham Packham. CRC Wessex Medical Oncology Unit, Cancer Sciences Division, School of Medicine, University of Southampton, Southampton General Hospital, Southampton, SO16 6YD, United Kingdom.

**Background:** BAG-1 is a multifunctional protein that binds a wide range of cellular targets including heat shock proteins and some nuclear hormone receptors. BAG-1 exists as three isoforms, BAG-1L, BAG-1M and BAG-1S. BAG-1L contains a nuclear localisation signal which is not present in the other isoforms and is predominantly localised in the cell nucleus.
**Aims:** To determine if BAG-1 expression is related to clinico-pathological features in breast cancer, and to determine if BAG-1 modulates oestrogen dependant transcription.
**Methods:** Following ethical committee approval (Southampton & S. West Hants LREC Submission: 159/00), BAG-1 expression in tumours from 138 patients with breast cancer treated with hormonal therapy was assessed by immunohistochemistry. We performed coimmunoprecipitation assays to analyse the interaction of BAG-1 isoforms with oestrogen receptor (ER) alpha and beta, and reporter gene assays to measure the effects of BAG-1 isoforms on oestrogen dependent transcription.
**Findings:** Nuclear but not cytoplasmic BAG-1 immunostaining was associated with improved survival in patients treated with hormonal therapy (Hazard Ratios: BAG-1 nuclear positive: 0.334 (95% Confidence Interval 0.138-0.809), BAG-1 nuclear negative: 1; P=0.015). There was also a strong inverse association between nuclear BAG-1 expression and tumour grade ($\chi^2$ test for trend=16.51, P<0.001). Nuclear BAG-1 expression was also moderately correlated with progesterone receptor (PgR) expression (r = 0.42) and ERα expression (r = 0.31). BAG-1L, but not BAG-1S or BAG-1M, interacted with both ERα and ERβ and increased oestrogen dependent transcription in ER positive MCF-7 breast cancer cells. BAG-1L overexpression stimulated transcription through both ERα and ERβ.
**Interpretation:** High levels of BAG-1L can increase responsiveness to oestrogens in breast cancer cells, and may be important in those cells for proliferation and survival. Similar to PgR and ERα status, BAG-1 may be a marker of responsiveness to hormonal therapy, via its direct effects on receptor function.

8.5

**ASSESSMENT OF A PUTATIVE ONCOGENE *ZNF217* WITHIN AMPLICON 20q BY MULTIPLEX QUANTITATIVE REAL-TIME PCR.**

Rooney PH[1*], Boonsong A[2], McFadyen M[1], McLeod HL[3], Cassidy J[2] and Murray GI[1] Departments of Pathology[1] and Medicine & Therapeutics[2], University of Aberdeen, Foresterhill, Aberdeen, AB25 2ZD UK. Department of Medicine[3], Washington University School of Medicine, 660 South Euclid Ave, Campus Box 8069, St. Louis, MO 63110-1093 USA.

Detection of gene amplification is a recognised process through which oncogenes can be identified. In this study we have coupled two powerful techniques, laser capture-microdissection and multiplex quantitative real-time PCR to quickly and accurately assess the gene copy number of candidate oncogene *ZNF217* in colon cancer. Several studies of colorectal cancer using comparative genomic hybridization (CGH) have identified chromosomal arm 20q, as the region most commonly gained in this tumour type[1]. In addition high density *FISH* mapping analysis of the 20q amplicon in colorectal cell lines within our laboratory has identified *ZNF217* to be contained within a region, 20q13.2 of high-level amplification. In this study the frequency and level of *ZNF217* amplification in 81 colon tumours was assessed by multiplex quantitative real-time PCR. Tumour cells were isolated from unfixed 10μm sections of original snap frozen tumour, using a PixCell II laser capture microdissection system (Arcturus Engineering). Tumor tissue was identified and removed by the laser to a microdissection cap. Approximately 500 laser pulses were taken per tumor (laser settings spot diameter set at 15μm, pulse duration 5 milliseconds and power 100mW). Following protein lysis with 20mg/ml proteinase K, DNA was assessed by Taqman PCR using the ABI7700 (PE Applied Biosystems). Multiplex quantitative real-time PCR was used to amplify internal control gene, *Beta globin* and the candidate gene, *ZNF217* simultaneously in the same well. For each case, *ZNF217* gene copy number was then calculated using the values of the mean $C_t$ of *ZNF217* (each case was assayed in triplicate) minus the $C_t$ of *Beta globin*. The tumour values of $C_t$ *ZNF217* – $C_t$ *Beta globin* were compared to those of normal diploid colon mucosa (n=10) and colorectal cell lines (n=6) for which *ZNF217* had been independently established using *FISH*, and assigned a specific copy number. Of the 81 tumours (42 Dukes' B and 39 Dukes' C) assessed, 18 were diploid, 15 had loss of gene copy and 48 contained some level of amplification at the *ZNF217* locus. Chi-square analysis found no significance in the distribution of *ZNF217* gene copy number between Dukes' B and Dukes' C tumours. In this study we found that ZNF217 amplification is a frequent event in colon cancer (48/81 tumours = 59.3%) and that the extent of its amplification varies markedly between tumours (range 3 - 13 copies).

1. Rooney PH, Murray GI, Stevenson DAJ, Haites NE, Cassidy J, McLeod HL. 1999 *Br J Cancer* **80**:862-873.

Acknowledgements: This research was funded by SHERT (Scottish Hospital Endowments Research Trust), GUHT (Grampian University Hospital Trust) and University of Aberdeen development trust.

8.6

**GENE EXPRESSION PROFILE ANALYSIS OF TUMOR-DERIVED CELL LINES OVEREXPRESSING THE CANDIDATE TUMOR SUPPRESSOR GENE, FIBULIN-1**

Zoë D. Kelly[*1], Jeremy L. Barth[2], Edward JP. Fox[1], Lisa M. Greene[1], Christian Knaak[2], Alison A. Murphy[1], W. Scott Argraves[2], and William M. Gallagher[1]. [1]Department of Pharmacology, Conway Institute of Biomolecular and Biomedical Research, Dublin 4, Ireland; [2]Department of Cell Biology and Anatomy, Medical University of South Carolina, Charleston, South Carolina, USA. Contact details: E-mail. william.gallagher@ucd.ie; Tel. +353-1-7161511; Fax +353-1-2692749.

Fibulin-1 is an extracellular matrix protein implicated as a suppressor of tumour cell adhesion, motility and growth. The mechanism by which fibulin-1 mediates these suppressive effects is unclear. Here, we used DNA microarray analysis to characterise a profile of genes whose expression is modulated by the two predominant fibulin-1 splice variants, fibulin-1C and D. The human breast tumour cell line MDA-MB-231 and the human lung fibrosarcoma cell line HT1080, both of which express little or no fibulin-1, were stably transfected to express fibulin-1C or D variants. Expression of the exogenous fibulin-1 variants was confirmed by immunoblot analysis of conditioned culture media. Using *Affymetrix* HuGeneFL arrays containing probe sets for 7,129 transcripts, gene expression profiles were determined in duplicate for both empty vector and fibulin-1 variant transfectants. Comparison of profiles obtained from empty vector- versus fibulin-1D-transfected MDA-MB-231 cells revealed 58 genes as being differentially expressed. Two hundred and thirty differentially expressed genes were identified in comparisons of empty vector- and fibulin-1D-transfected HT1080 cells. Furthermore, 28 genes were identified in comparisons of empty vector- and fibulin-1C-transfected HT1080 cells. Analysis of these profiles revealed evidence of fibulin-1-dependent effects on gene expression that included variant-specific effects. For example, expression of thrombospondin-1 increased by ~2 fold in fibulin-1D expressing cells (both MDA-MB-231 and HT1080), but decreased by 5.0 fold in fibulin-1C expressing HT1080 cells. In contrast, collagen alpha 1 type IV expression increased > 3.0 fold in cells expressing either fibulin-1 variant. OB-cadherin (cadherin 11), a calcium-dependent cell adhesion protein, was differentially expressed in fibulin-1 transfectants as compared to empty vector transfectants. Specifically, OB-cadherin expression was increased in the fibulin-1D-transfected MDA-MB-231 cells (+20.8 fold) and decreased in both the fibulin-1C (-9.4 fold) and D (-5.4 fold) transfected HT1080 cells. Transcripts for two other genes, ARPC4 (-3.8 fold) and RhoGDIbeta (+2.0 fold), were differentially expressed between the HT1080 fibulin-1C and empty vector transfectants. Based on these findings, it is hypothesised that fibulin-1 acts to (a) inhibit ARPC4 gene expression, with a consequent negative impact on cell motility, and (b) stimulate Rho GDIbeta gene expression, which in turn inactivates Rho-GTPase-dependent pathways, resulting in decreased cellular motility and tumourigenic reversion. [Supported by the Health Research Board - Ireland, Enterprise Ireland and

© 2002 Cancer Research UK

the Association for International Cancer Research (WMG) and NIH grant HL52813 (WSA)].

### 8.7

### THE RELATIONSHIP BETWEEN *IN VITRO* OXIDATIVE DAMAGE FORMATION AND MUTATION AT NUCLEOTIDE RESOLUTION IN *P53.*

KJ Bowman[1*], Y Guichard[1], L Langford[1], TR O'Connor[3], MN Routledge[4], GB Scott[5], PA Burns[5], GDD Jones[1], [1]Department of Oncology, [2]MRC Toxicology Unit, Hodgkin Building, University of Leicester, Leicester, UK, [3]Beckman Research Institute, Duarte, CA, USA, [4]Molecular Epidemiology Unit, [5]Department of Pathology, University of Leeds, Leeds, UK.

For certain carcinogens the pattern of DNA adducts produced in *p53* has been shown to be related to the pattern of *p53* mutation in associated human tumours; establishing causative links between the agents and cancers induced. Similarly, a positive correlation between patterns of *p53* oxidative damage and mutation could help establish the cancer relatedness of oxidative DNA damage. Until recently it was not possible to compare induced damage with mutation in *p53 in vitro*, as no suitable mutation assay was available. However, in the present study we have compared the distribution of induced oxidative damage with the distribution of induced mutation *in vitro* in human *p53* using LMPCR and a developed plasmid-based *p53* forward mutation assay, respectively. Briefly, LMPCR permits the detection of rare DNA breaks along single copy genes at nucleotide resolution in the full human genome, and combining this technique with damage recognising *E. coli* Nth and Fpg proteins allows for mapping of oxidative base lesions. In the mutation assay, a plasmid bearing a full *c*-DNA copy of human *p53* is damage-treated *in vitro*, then transformed into yeast cells containing an *ADE2* gene under the control of a *p53* protein-responsive promoter. Transformed colonies containing mutant p53 protein are unable to express the *ADE2* gene and accumulate a red metabolite. The red colonies are picked, grown and the human *p53* amplified and sequenced. Using LMPCR we have mapped $Cu+H_2O_2$-induced oxidative damage along the transcribed strand of exons 5 and 7 of *p53* in isolated human DNA, and have compared this with the distribution of mutations induced by $Fe+H_2O_2$+ascorbate reactions in the plasmid *c*-DNA *p53* exon 5 and 7 sequences. Even with the different gene targets and damaging agents used, there is a marked degree of similarity between the distribution of damage and mutation, particularly for exon 5 where the regions of greatest damage (exclusively manifest at guanines in codons 142, 149-156, 177-178) strongly correlate with the locations of highest mutation frequency (predominately GC/TA transversions and GC/AT transitions at codons 143, 151-154, 177). The similarity between the Cu-dependent damage and Fe-dependent mutation spectra is in line with metal-ion dependent peroxide-induced damage being more sequence specific than metal specific[1]. Future studies directly comparing the same gene-targets and damaging agents, will allow us to rigorously address which damaged sites are critical to mutation, and any correlation with the reported *in vivo* mutations in human tumours will greatly increase relevance of our findings to human carcinogenesis.

1. Rodriguez *et. al.* (1997) *Cancer Research* **57**, 2394.

### 8.8

### GENETIC LESIONS OCCUR IN PLASMA DNA OF PATIENTS WITH LUNG CANCER AND CHRONIC RESPIRATORY DISEASES (CRD).

S Khan[*1], J Coulson[2] & P J Woll1[1] , Prof J Britton,[1] [1]CRC Department of Clinical Oncology, University of Nottingham, City Hospital, Nottingham NG5 1PB, UK, [2] Department of Physiology, University of Liverpool.

**BACKGROUND.** Tumours arise through an accumulation of genetic and epigenetic alterations. In lung cancer, these include deletions on chromosomes 3p, 8p, 9p, 5q, Rb, 17p (resulting in loss of heterozygosity, LOH) and p53 mutations. This study compared genetic alterations in plasma DNA and lymphocyte (control) DNA from patients with lung cancer and CRD. **METHODS.** 5mls blood was collected in sodium citrate, ( full ethical written consent was obtained prior to collection). Plasma and cells were separated by centrifugation, then stored at -80$^0$C. DNA was extracted using QIAmp Blood DNA Kit (Qiagen). PCR was performed using $P^{32}$-labelled primer pairs to assess LOH. The products were separated on 6% polyacrylamide gel and autoradiographed. **RESULTS.** 166 patients were studied, 53 lung cancer, 113 CRD. Following initial analysis of 20 markers in 32 lung cancer samples, three markers were selected (D3S1300(3p14.2), D8S201(8pter-p23), D3S1560(3pter-p24.2)). Patient characteristics and results are shown in the table. **DISCUSSION.** Using a combination of all 3 markers, an overall detection rate of LOH/MI of 70% is achieved in lung cancer patients, and 73% in CRD patients. There is a significant difference in the number of genetic lesions between the lung cancer group and CRD group ($p< 0.001$). There is limited data on genetic abnormalities in patients with CRD who are at risk of developing lung cancer. A majority of these patients were smokers. Results suggest that smokers with chronic lung disease have frequent genetic alterations on chromosomes 3 and 8p.

Table. Patient characteristics and LOH/MI Detection Rates

| Patient Group | CRD | Lung Cancer |
|---|---|---|
| Number | 113 | 53 |
| Age, median (range) | 62 (31-87) | 66 (37-82) |
| Gender, M/F | 78/35 | 32/21 |
| Disease | COPD, 32<br>Interstitial Lung Disease, 16<br>Bronchiectasis, 13<br>Occupational, 7<br>Asthma, 13<br>Others, 32 | NSCLC, 20<br>SCLC, 33 |
| LOH/MI Detection: Number (%) | | |
| D3S1300 | 29 (26%) | 11 (21%) |
| D8S201 | 24 (21%) | 28 (53%) |
| D3S1560 | 67 (59%) | 27 (51%) |
| Any positive | 73% | 70% |

© 2002 Cancer Research UK

